# Date on identification of flavonoids in *Plumula nelumbinis* by UPLC-ESI-QTOF-MS and antioxidant activity from 13 habitats in China

**DOI:** 10.1016/j.dib.2018.09.114

**Published:** 2018-10-03

**Authors:** Junxia Zheng, Wenyue Tian, Chao Yang, Weipeng Shi, Peihong Cao, Jiatang Long, Luomin Xiao, Yang Wu, Jizheng Liang, Xiaobin Li, Suqing Zhao, Kun Zhang, Hui Zhi, Pinghua Sun

**Affiliations:** a Institute of Natural Medicine and Green Chemistry, School of Chemical Engineering and Light Industry, Guangdong University of Technology, Guangzhou 510006, PR China; b School of Pharmaceutical Science, Guangzhou University of Chinese Medicine, Guangzhou 510006, PR China; c Guangdong Province Key Laboratory of Pharmacodynamic Constituents of TCM and New Drugs Research, College of Pharmacy, Jinan University, Guangzhou 510632, PR China; d School of Chemistry and Environment Engineering, Wuyi University, Jiangmen 529020, PR China

## Abstract

*Plumula nelumbinis* is a well-known health food and a traditional Chinese medicine, is used in many countries around the world. For its pharmacological properties are related to its chemical composition, a ultra-performance liquid chromatography electrospray ionization quadrupole time-of-flight mass spectrometry (UPLC-ESI-QTOF-MS) method was used to identify the flavonoids in *P. nelumbinis*. The first set of data shows the MS-MS Spectrograms of 12 flavonoid standards and 38 flavonoids detected in the *P. nelumbinis* from Xiangtan, Hunan province. The second set of data shows the total flavonoids content and the antioxidant activity of total flavonoid in *P. nelumbinis* from 13 habitats. The antioxidant activity were accomplished with 1,1-diphenyl-2-picrylhydrazyl (DPPH), oxygen radical absorbance capacity (ORAC) and ferric reducing ability of plasma (FRAP) assays.

**Specifications table**

**Table:** 

Subject area	Chemistry, Biology
More specific subject area	Natural product chemistry
Type of data	Table, figures
How data was acquired	Thirty-eight flavonoid compounds in *Plumula nelumbinis* were identified by ultra-performance liquid chromatography electrospray ionization quadrupole time-of-flight mass spectrometry method. Total flavonoids contents were determined using colorimetric methods. The variations in concentration of flavonoids and antioxidant activity in plants from different habitats in China were investigated using three assays,as 1,1-diphenyl-2-picrylhydrazyl (DPPH) assay, oxygen radical absorbance capacity assay (ORAC) and ferric reducing ability of plasma assay (FRAP).
Data format	Raw, Analyzed
Experimental factors	Dried *Plumula nelumbinis* extracted three times with 2.4 L of 80% EtOH-H_2_O for 2 h each time, dissolved in 0.1% HCl, then loaded on the pretreated D001 cation exchange resin and AB-8 macroporous resin chromatography. The supernatants were filtered through a 0.22-µm membrane filter before injection into the UPLC-ESI-QTOF-MS system for analysis.
Experimental features	Identification the flavonoids in *Plumula nelumbinis* by UPLC-ESI-QTOF- MS and the total flavonoids content, antixiodant capacity from thirteen different habitats were determined.
Data source location	Thirteen locations, China [1]
Data accessibility	The data are available with in this article
Related research article	Identification of Flavonoids in *Plumula nelumbinis* and evaluation of their antioxidant properties from different habitats (in press)

**Value of the data**

● Method and data can be used to identify natural flavonoids by UPLC-ESI-QTOF-MS.
● The chromatographic and mass spectrometric data can be used for comparison with other studies performed on *Plumula nelumbinis*, then serve as a benchmark for other researchers to elucidate the constituents of *Plumula nelumbinis*.
● The flavonoid contents and the antioxidant activity data will provide a valuable reference for studies comparing the chemical and pharmacological effects of *Plumula nelumbinis*.

## 1 Data

**Fig. 1:**
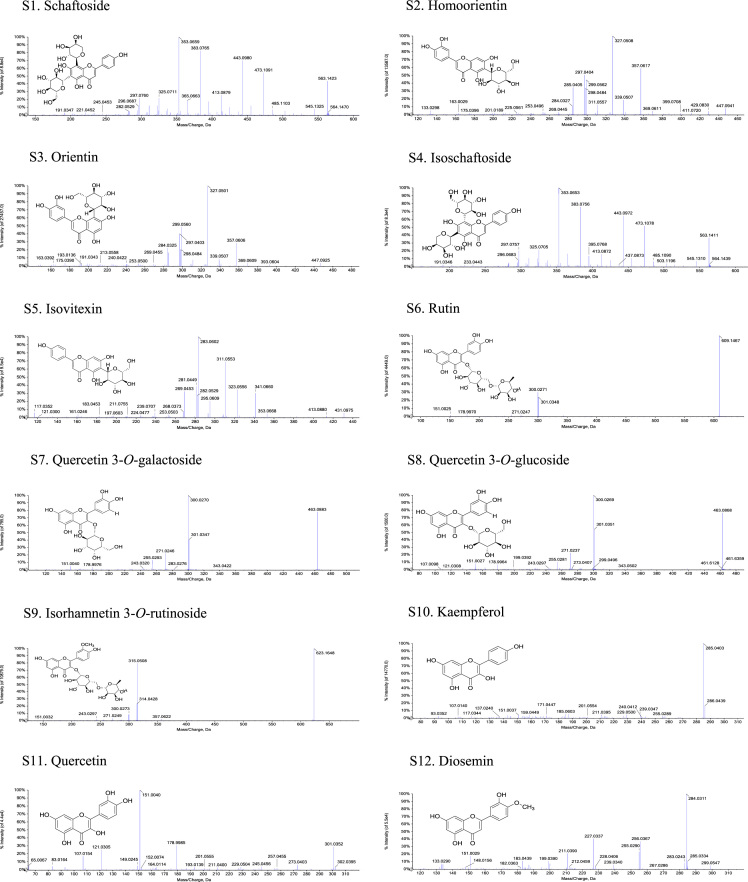
The structures and MS-MS Spectrograms of 12 flavonoid standards. (S: standard).

**Fig. 2:**
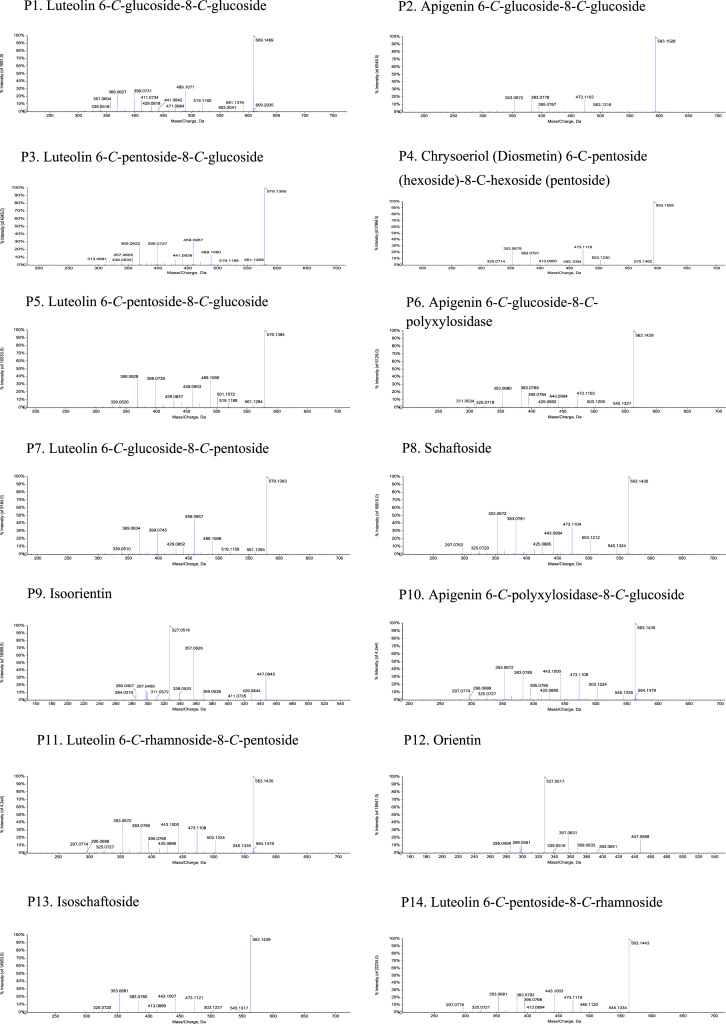

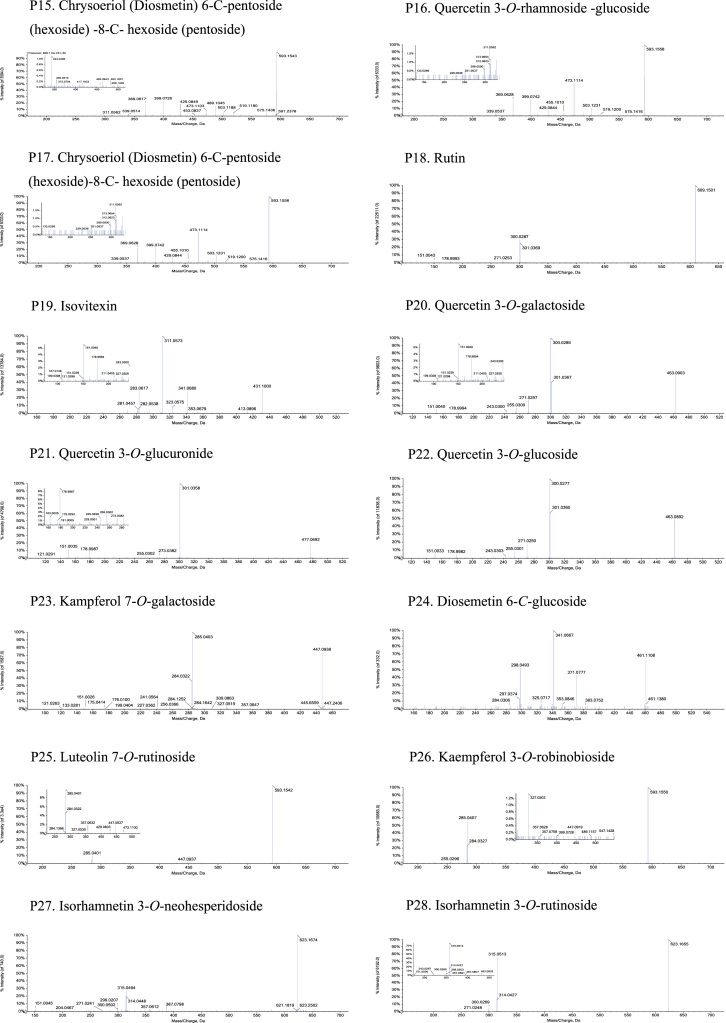

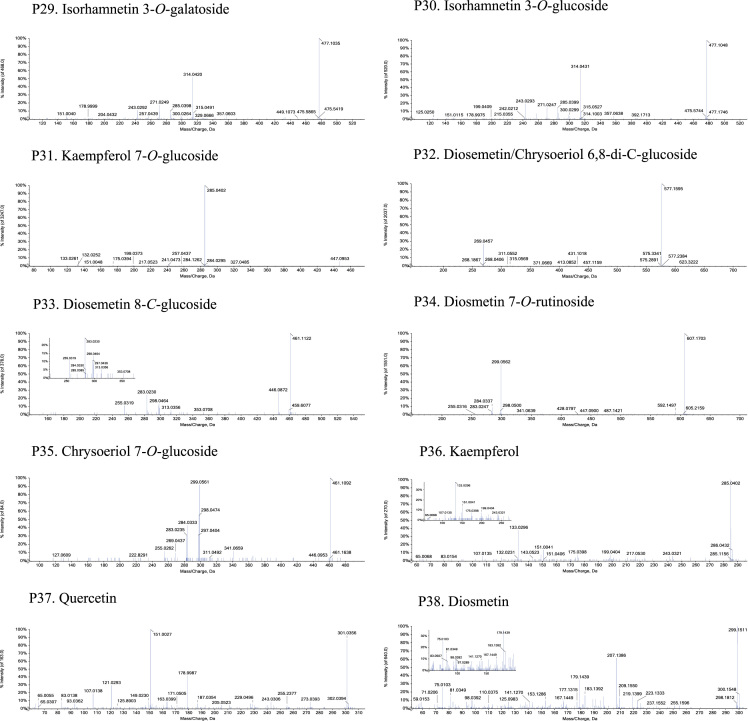
The MS-MS Spectrograms of 38 flavonoids detected in the *Plumula nelumbinis* from Xiangtan, Hunan province. (P: peak).

The structures and MS-MS spectrograms of 12 flavonoid standards are provided in Fig. 1. Data in Fig. 2 presents the MS-MS spectrograms of 38 flavonoids detected in the *Plumula nelumbinis* from Xiangtan, Hunan province. Data in Table 1 includes the total flavonoids content and antioxidant capacity of *P. nelumbinis* obtained from 13 different habitats.

**Table 1:** Total flavonoids content and antioxidant capacity of *Plumula nelumbinis* obtained from different habitats, cited on a dry weight basis.

sample	Flavonoids	DPPH	FRAP	ORAC
	(µg RE^a^/mg DW)	IC_50_ (μg/mL)	(mmol Fe(II)/g DW)	(µmol TE^a^/mg DW)
Hunanxiangtan	106.32 ± 0.23^b^	71.28 ± 0.62	2.39 ± 0.025	1.60 ± 0.002
Jiangxiguangchang	85.41 ± 0.46	450.21 ± 1.93	1.50 ± 0.039	1.04 ± 0.001
Jiangxishicheng	97.11 ± 0.46	753.93 ± 2.80	2.68 ± 0.079	1.99 ± 0.002
Shandongweihu	87.24 ± 1.00	82.96 ± 0.76	1.63 ± 0.070	1.56 ± 0.003
Shandongheze	117.82 ± 0.35	130.53 ± 0.86	3.28 ± 0.075	2.71 ± 0.001
Zhejianghangzhou	74.63 ± 0.61	362.64 ± 1.74	1.71 ± 0.049	1.70 ± 0.002
Fujiannanping	79.37 ± 0.35	> 1000	1.13 ± 0.017	1.07 ± 0.004
Shanxiweinan	97.11 ± 0.96	510.62 ± 3.02	0.66 ± 0.002	0.38 ± 0.003
Hubeihonghu	60.79 ± 0.48	421.18 ± 2.12	0.52 ± 0.000	0.52 ± 0.004
Anhuibozhou	44.51 ± 0.58	851.86 ± 2.65	0.36 ± 0.004	0.28 ± 0.003
Yunnanwenshan	63.62 ± 0.69	> 1000	0.34 ± 0.005	0.27 ± 0.006
Zhejiangwuyi	74.25 ± 0.81	432.25 ± 1.68	0.64 ± 0.002	0.76 ± 0.005
Fujianfuzhou	76.31 ± 0.26	456.76 ± 0.98	0.60 ± 0.006	0.87 ± 0.008

a RE is the abbreviation of rutin equivalents, TE is the abbreviation of Trolox equivalents, DW is the abbreviation of dry weight of sample;

b IC_50_ is the concentration of sample required to scavenge 50% of DPPH radicals;^c^Each value is expressed as mean ± S.D. (*n* = 3).

## 2 Experimental design, materials, and methods

### 2.1 Chemicals and reagents

A detailed description of chemicals and reagents is provided in Ref. [1].

### 2.2 Plant materials preparation, extraction and isolation

The *P. nelumbinis* were collected during August, 2017 from thirteen locations in China, the Coordinates and locations of these 13 varieties are provided in Ref. [1].

The *P. nelumbinis* was extracted with 80% EtOH-H_2_O, isolated by D001 cation exchange resin and AB-8 macroporous resin chromatography as described in Ref. [1].

### 2.3 HPLC-ESI-QTOF-MS parameter

A triple TOFTM 5600+ mass spectrometer (AB Sciex, Foster City, CA) coupled with a Shimadzu Prominence UPLC system (Nexera UHPLC LC-30A, Kyoto, Japan) was used to analysis samples. The condition of LC system and mass spectrometric detection were selected as described in Ref. [1]. The Peak View analysis software was used for data analysis.

### 2.4 Total flavonoid content, DPPH, ORAC and FRAP determination

The total flavonoid content, DPPH free radical scavenging activity, Oxygen radical absorbance capacity and Ferric reducing antioxidant power of *P. nelumbinis* were determined according to the methods in Ref. [1].
